# Radiomics-driven neuro-fuzzy framework for rule generation to enhance explainability in MRI-based brain tumor segmentation

**DOI:** 10.3389/fninf.2025.1550432

**Published:** 2025-04-17

**Authors:** Leondry Mayeta-Revilla, Eduardo P. Cavieres, Matías Salinas, Diego Mellado, Sebastian Ponce, Francisco Torres Moyano, Steren Chabert, Marvin Querales, Julio Sotelo, Rodrigo Salas

**Affiliations:** ^1^PhD Program in Health Sciences and Engineering, Universidad de Valparaíso, Valparaíso, Chile; ^2^Faculty of Engineering, School of Biomedical Engineering, Universidad de Valparaíso, Valparaíso, Chile; ^3^Center of Interdisciplinary Biomedical and Engineering Research for Health - MEDING, Universidad de Valparaíso, Valparaíso, Chile; ^4^Millennium Institute for Intelligent Healthcare Engineering (iHealth), Santiago, Chile; ^5^Faculty of Medicine, School of Medical Technology, Universidad de Valparaíso, Valparaíso, Chile; ^6^Servicio de Imagenología, Hospital Carlos van Buren, Valparaíso, Chile; ^7^Centro para la Investigación Traslacional en Neurofarmacología (CITNE), Universidad de Valparaíso, Valparaíso, Chile; ^8^Departamento de Informática, Universidad Técnica Federico Santa María, Santiago, Chile

**Keywords:** radiomics, neuro-fuzzy systems, decision rules, brain tumor segmentation, Explainable Artificial Intelligence, magnetic resonance imaging, deep learning

## Abstract

**Introduction:**

Brain tumors are a leading cause of mortality worldwide, with early and accurate diagnosis being essential for effective treatment. Although Deep Learning (DL) models offer strong performance in tumor detection and segmentation using MRI, their black-box nature hinders clinical adoption due to a lack of interpretability.

**Methods:**

We present a hybrid AI framework that integrates a 3D U-Net Convolutional Neural Network for MRI-based tumor segmentation with radiomic feature extraction. Dimensionality reduction is performed using machine learning, and an Adaptive Neuro-Fuzzy Inference System (ANFIS) is employed to produce interpretable decision rules. Each experiment is constrained to a small set of high-impact radiomic features to enhance clarity and reduce complexity.

**Results:**

The framework was validated on the BraTS2020 dataset, achieving an average DICE Score of 82.94% for tumor core segmentation and 76.06% for edema segmentation. Classification tasks yielded accuracies of 95.43% for binary (healthy vs. tumor) and 92.14% for multi-class (healthy vs. tumor core vs. edema) problems. A concise set of 18 fuzzy rules was generated to provide clinically interpretable outputs.

**Discussion:**

Our approach balances high diagnostic accuracy with enhanced interpretability, addressing a critical barrier in applying DL models in clinical settings. Integrating of ANFIS and radiomics supports transparent decision-making, facilitating greater trust and applicability in real-world medical diagnostics assistance.

## 1 Introduction

The incidence rate of primary brain and other central nervous system (CNS) tumors has increased, likely due to advances in diagnostic technologies, revisions in classification systems, and increased access to diagnostic imaging devices worldwide (Louis et al., 2021; de Robles et al., 2015; Low et al., 2022). These tumors represent a significant public health concern due to their high rates of mortality and disability, particularly malignant forms, which account for 1.4% of all cancers and 2.3% of cancer-related deaths (McNeill, 2016). Approximately 30,000–35,000 new cases are expected yearly in the United States alone. In adults, the most frequently observed sites of non-brain malignancies were mammary, prostatic, colorectal, and cutaneous melanoma (Neff et al., 2023; Sancar et al., 2017). Notably, BT constitute the second most common cancer type in pediatric populations (Zhang et al., 2017). Treatment strategies vary depending on tumor type and location, with glioblastomas and meningiomas being the most common malignant and non-malignant tumors, respectively (Low et al., 2022; Chabert et al., 2024). A significant challenge in the management of BT is their infiltrative growth pattern. Unlike many other cancers, which may exhibit defined margins, glioblastomas can integrate into surrounding neural tissue, making complete surgical resection challenging (Ban et al., 2021). As tumors have different radiological characteristics, edema limits the correct definition of boundaries to analyze specific affected regions using medical imaging, making treatment planning difficult (Csaholczi et al., 2020; Khan and Park, 2024).

Diagnosis of BT traditionally depends on the expertise of neuro-radiologists, who perform detailed clinical analyses and thorough evaluations of imaging results. However, the global shortage of specialized professionals makes this process time-consuming and resource-intensive (Ali et al., 2022). Medical imaging techniques are an essential tool for visualization and diagnosis of anatomical structures and physiological processes. The most commonly used are MRI, Computed Tomography (CT), Positron Emission Tomography (PET), and X-rays (Bahkali and Semwal, 2021; Hussain et al., 2022). In contrast to previous techniques, MRI is considered the reference method for diagnosing and characterizing BT due to its superior anatomical resolution and its ability to differentiate soft tissue structures without using ionizing radiation (Song et al., 2016; Zhou et al., 2022). It presents an optimal modality for patients who require repeated examinations, such as pediatric cases or individuals who require long-term follow-up (Iradat et al., 2024; Jamieson et al., 2013). In response, Artificial Intelligence (AI) models have emerged as powerful tools to assist in tumor detection and classification using MRI (Dixit and Thakur, 2023; Bouhafra and El Bahi, 2024; Rasheed et al., 2023). Machine Learning (ML) and Deep Learning (DL) techniques have been widely adopted for BT detection, harnessing MRI scans to provide fast and accurate predictions (Tabatabaei et al., 2023; Özkaraca et al., 2023; Mohanty et al., 2024). These AI-driven approaches are increasingly helping medical professional improve patient care by improving diagnostic efficiency and accuracy (Cè et al., 2023; Veloz et al., 2011). Nevertheless, it is imperative to consider the limitations associated with implementing DL techniques in healthcare. The requirement for extensive annotated datasets presents a significant impediment (Vrochidou et al., 2023). The computational resources necessary to process and analyze these datasets are substantial, often necessitating a high-performance computing infrastructure that may not be easily accessible in healthcare environments (Zhang et al., 2024; Filippini et al., 2023). This challenge is exacerbated by the labor intensive nature of data annotation, which is crucial for training the DL model, but can be prohibitively resource intensive (Mitchell et al., 2021). The advent of transfer learning techniques has further accelerated progress, enabling AI models to achieve high accuracy even with limited datasets, while significantly reducing training time (Alnemer and Rasheed, 2021). The performance of DL algorithms is heavily dependent on the availability of large amounts of training data. However, healthcare data is often limited in volume and quality due to factors such as patient sparsity, variability in medical practices, and strict privacy regulations (Chen et al., 2019). Other studies, such as Chen et al. (2021), discuss the challenge of generalizing DL models trained on limited datasets, and highlight the importance of having diverse training sets to achieve robust performance across different settings or demographics of patients (Chen et al., 2021). Variations in data acquisition protocols between institutions can lead to discrepancies in image characteristics, affecting model performance. DL offers advantages over traditional ML approaches by automatically extracting high-level features from input data. It demonstrates efficacy in complex medical imaging tasks such as disease classification and tumor segmentation (Torres-Velázquez et al., 2020). However, the implementation of DL in medical settings remains challenging due to generalization issues (Yoon et al., 2023). Despite these advancements, a persistent challenge lies in the lack of transparency in AI methodologies (Burkart and Huber, 2021). This opacity undermines trust in AI-driven systems and raises ethical concerns among healthcare professionals, posing a significant barrier to widespread clinical adoption.

Explainable Artificial Intelligence (XAI) addresses these problems by designing transparent and interpretable models, i.e., models that can provide explanatory information in a form accessible to humans (Schiavon et al., 2023). The goal of XAI is to help humans trust AI systems more and thus enable better human-expert machine interaction (Ugalde et al., 2023). To explain the machine learning models currently being used for BT classification and segmentation, some methods have been proposed, such as class activation maps (CAMs) (Schiavon et al., 2023), attention maps (Tehsin et al., 2024), model-agnostic methods such as SHAP (SHapley Additive exPlanations) (Ahmed et al., 2023) or interpreting key features using radiomics (Afshar et al., 2019; Ponce et al., 2024). Such approaches demonstrate the potential to provide quantitative and reproducible metrics of interpretation and validation for medical imaging data (Zhang X. et al., 2022).

Most of the current explainability methods developed for BT classification algorithms do not provide deeper insights into their operational mechanisms and tend to produce heatmaps that simply determine the relevance of specific input features or variables. Although these approaches are beneficial, they are insufficient in providing a complete explanation of the rationale behind the predictions. Through this method, we intended to propose an interpretable decision rule based on fuzzy logic which further supports the prediction process and provides us insights based on that prediction. In the leading approach, MRI scans are preprocessed and tumor regions are segmented using 3D U-Nets convolutional neural networks (CNN) (Cavieres et al., 2023). From there, radiomic characteristics about compression, texture, and pixel values are extracted (Ponce et al., 2024). In order to increase their efficiency, dimensionality reduction methods are used to keep only the most discriminative features. Finally, it trains an Adaptive Network-based Fuzzy Inference System (ANFIS) to classify BT and obtain a set of decision rules that facilitate clinical interpretability (Querales et al., 2023; Allende-Cid et al., 2016). This method ensures the performance of the assisted diagnosis is maintained at a high level, and along with this, can provide explanations in an accurate and interpretable way, significantly enhancing the clinical reliability and applicability of AI therapy solutions.

This research is structured as follows. The Section 2 provides a comprehensive review of related work, highlighting previous studies and current methodologies used in BT detection and classification, as well as exploring strategies for the explainability of the models and the interpretation of their predictions. The Section 3 details the methodology used, including the main steps such as database, pre-processing, segmentation, feature extraction and classification. The results, presented in Section 4, highlight the performance and explainability capabilities of the proposed model, underlining its advantages over traditional approaches. Section 5 elaborates on the results' analysis, highlighting the proposal's novelty and its contribution to assisted diagnosis through detailed tumor characterization. Finally, Section 6 summarizes the main findings and suggests possible future research lines to improve the clinical applicability and interpretability of the models.

## 2 Related works

The integration of Explainable Artificial Intelligence (XAI) into BT classification and segmentation has become a critical area of research, addressing the dual challenge of improving the accuracy of diagnostic assistance while ensuring model interpretability. For example, studies such as Ullah et al. (2024) and Saeed et al. (2024) focus on BT segmentation and classification using advanced architectures like DeepLabV3+. Ullah et al. (2024) introduces a comprehensive two-component framework that incorpores Bayesian optimization for hyperparameter tuning. The study leverages models such as the Inverted Residual Bottleneck to enhance classification performance and uses Local Interpretable Model-Agnostic Explanations (LIME) to provide insights into predictions. Similarly, Saeed et al. (2024) integrates self-attention modules into the DeepLabV3+ architecture, combining features extracted from CNN architectures like Darknet53 and MobileNetV2 with a Bayesian optimized Support Vector Machine (SVM). Grad-CAM techniques are employed to visualize heatmaps, offering clinicians a clearer understanding of model predictions. In contrast, other studies, such as Selvapandian and Manivannan (2018) and Schiavon et al. (2023), explore alternative methodologies for BT classification. Selvapandian and Manivannan (2018) employ morphological operations for glioma segmentation, texture analysis, and classification using ANFIS, with good performance. Meanwhile, Schiavon et al. (2023) highlights the role of CNN architectures in classification, using XAI techniques like Grad-CAM and CAMs to interpret predictions. This underscores the importance of identifying critical features in medical images, offering valuable insights for clinical decision-making.

Recent studies have explored machine learning (ML) approaches for brain tumor (BT) classification using MRI. Various algorithms have been evaluated, including k-Nearest Neighbors (k-NN), Random Forest (RF), Linear Discriminant Analysis (LDA), Decision Trees (DT), Logistic Regression (LR), and Multilayer Perceptron (MLP) (Çınarer and Emiroğlu, 2019; Ferdous et al., 2021; Kale et al., 2024; Yin and Wang, 2024). For instance, Kale et al. (2024) analyzed MRI brain scans to classify tumor and non-tumor tissue using LR, MLP, and RF, achieving accuracy rates of 96%, 95%, and 96%, respectively. Similarly, Sahoo et al. (2020) assessed the effectiveness of various ML algorithms for BT classification, reporting that k-NN achieved an average detection accuracy of 96.4%. For multi-class classification, Saraswathi and Gupta (2019) demonstrated that RF achieved 88.7% accuracy in distinguishing between different tumor types. Additionally, Latif et al. (2018) proposed an enhanced classification methodology incorporating hybrid statistical and wavelet features, achieving 96.72% accuracy for high-grade gliomas and 96.04% for low-grade gliomas using MLP. These studies illustrate the successful application of ML classification methods for BT detection and segmentation. Furthermore, they provide a benchmark for evaluating the proposed framework, validating its comparative performance against existing classification models.

Additional studies, such as Afshar et al. (2019) and Padmapriya and Devi (2024), explore the interpretability of alternative architectures in BT analysis. Afshar et al. (2019) focuses on Capsule Networks, employing techniques such as maximization of activation to bridge the gap between automated classification and human-understandable reasoning, offering a novel perspective on interpretability. Similarly, Padmapriya and Devi (2024) develops a computer-aided diagnostic (CAD) system that uses Grad-CAM to visualize critical regions on MRI scans, improve clinician confidence by providing intuitive visual explanations. In addition, Benyamina et al. (2022) advances the explainability of deep transfer learning models by leveraging SHAP values to clarify the decision-making process in tumor classification tasks. By analyzing diverse MRI datasets, this study underscores the persistent challenges of balancing high classification accuracy with the need for interpretable AI models, highlighting the importance of transparent decision-making in clinical applications.

Although several studies have used ANFIS for the classification of BT and have achieved high accuracy, they often lack interpretability, limiting their clinical applicability. For example, Shanmugam and Surampudi (2022) achieved high accuracy in BT classification using ANFIS but failed to provide mechanisms to explain how radiomic features influenced the decisions. Similarly, Anitha et al. (2023) combined the curvelet transform with ANFIS for the detection and segmentation of meningioma, achieving high accuracy while partially addressing interpretability by linking specific tumor features to the classification results. Kalam et al. (2021) presented an optimized ANFIS classifier with improved precision, recall and sensitivity to detect meningiomas, gliomas, and pituitary tumors, but the contributions of individual characteristics to the decision-making process were not clarified. Likewise, Kshirsagar et al. (2022) used Gray Level Co-occurrence Matrix (GLCM) features with ANFIS to classify BT as normal, benign, or malignant, achieving high accuracy, yet without addressing the interpretability of the decision-making process. To our knowledge, there is still a gap in developing explainability mechanisms that clarify how Artificial Intelligence (AI) techniques combine and prioritize the most relevant features for classification. To address this, this work proposes a methodology for constructing interpretable rules that elucidate the key elements used to determine whether tissue is cancerous.

## 3 Materials and methods

### 3.1 Database

This study assesses the performance of a model designed for interpretability in classifying BT, using the BraTS2020 dataset. The dataset, available at Menze et al. (2015), is a benchmark resource for BT segmentation and consists of multi-contrast MRI scans. It includes four imaging sequences: native T1-weighted, post-contrast T1-weighted (T1ce), T2-weighted and Fluid Attenuated Inversion Recovery (FLAIR), collected from 366 patients. Each MRI scan has a resolution of 240 × 240 × 155 voxels and is accompanied by manually segmented masks and expert-validated tumor segmentation labels. These labels differentiate between three key regions: enhancing tumor (*ET*), tumor core (*TC*), and edema (*E*).

To prepare the images from the BraTS2020 database for segmentation, several pre-processing steps were applied. First, pixel values were normalized to a range of 0–1, minimizing intensity variations caused by differences in imaging equipment. The images were then resized to a uniform resolution of 128 × 128 × 128 voxels to ensure consistency throughout the data set. Resizing ensures uniformity in image dimensions, enhancing consistency in model input for robust training (Zubair Rahman et al., 2024). The process includes cropping dark areas from the images while preserving the brain region. This approach not only mitigates class imbalance but also reduces computational costs, thereby improving the model's efficiency (Das et al., 2022). Quadratic interpolation was applied to adjust the images, while nearest-neighbor interpolation was used for the segmentation masks to preserve their discrete nature. Non-informative regions, such as dark areas or white spaces around the edges, were removed to focus on the regions of interest. For model training, 80% of the dataset was allocated, with the remaining 20% reserved for testing.

#### 3.1.1 Class imbalance adjustments

Class imbalance is a significant challenge in data classification, as it directly impacts model performance and parameter optimization (Luque et al., 2019). Imbalanced datasets impede the learning process, particularly for minority classes, often resulting in their misclassification (Rezvani and Wang, 2023). Although random undersampling and oversampling are commonly used baseline methods, our approach prioritizes undersampling to achieve a more effective balance between majority and minority classes. This strategy is especially useful for our dataset, where one class is disproportionately over-represented (Rezvani and Wang, 2023). Although oversampling can be advantageous in certain scenarios, it carries the risk of overfitting by duplicating examples from minority classes, which can reduce the generalizability of the model (Fernández et al., 2018; Estabrooks et al., 2004).

The label adjustment was applied exclusively to the training set. This adjustment was performed on the basis of two experimental criteria. In Experiment 1, the focus was on distinguishing between healthy tissue and whole tumor tissue, while in Experiment 2, the differentiation extended to three categories: healthy tissue, tumor core, and edema. For both experiments, the minority class served as the reference, and adjustments were made independently for each class to address imbalances. To emphasize the different outcomes of these adjustments, Table 1 presents the results for both experimental setups, highlighting the impact of the adjustments on class distribution.

**Table 1:** Adjustments to handle the imbalance in the database.

**Experiment**	**Labels**	**Training process samples**		**Validation samples**
		**Initial number**	**Undersampled**	
Experiment 1	Healthy tissue	1,077,737	159,558	213,835
	Whole tumor	159,558	159,558	34,689
Experiment 2	Healthy tissue	1,077,737	75,236	213,835
	Tumor core	84,322	75,236	18,958
	Edema	75,236	75,236	15,731

### 3.2 Proposed framework

**Figure 1:**
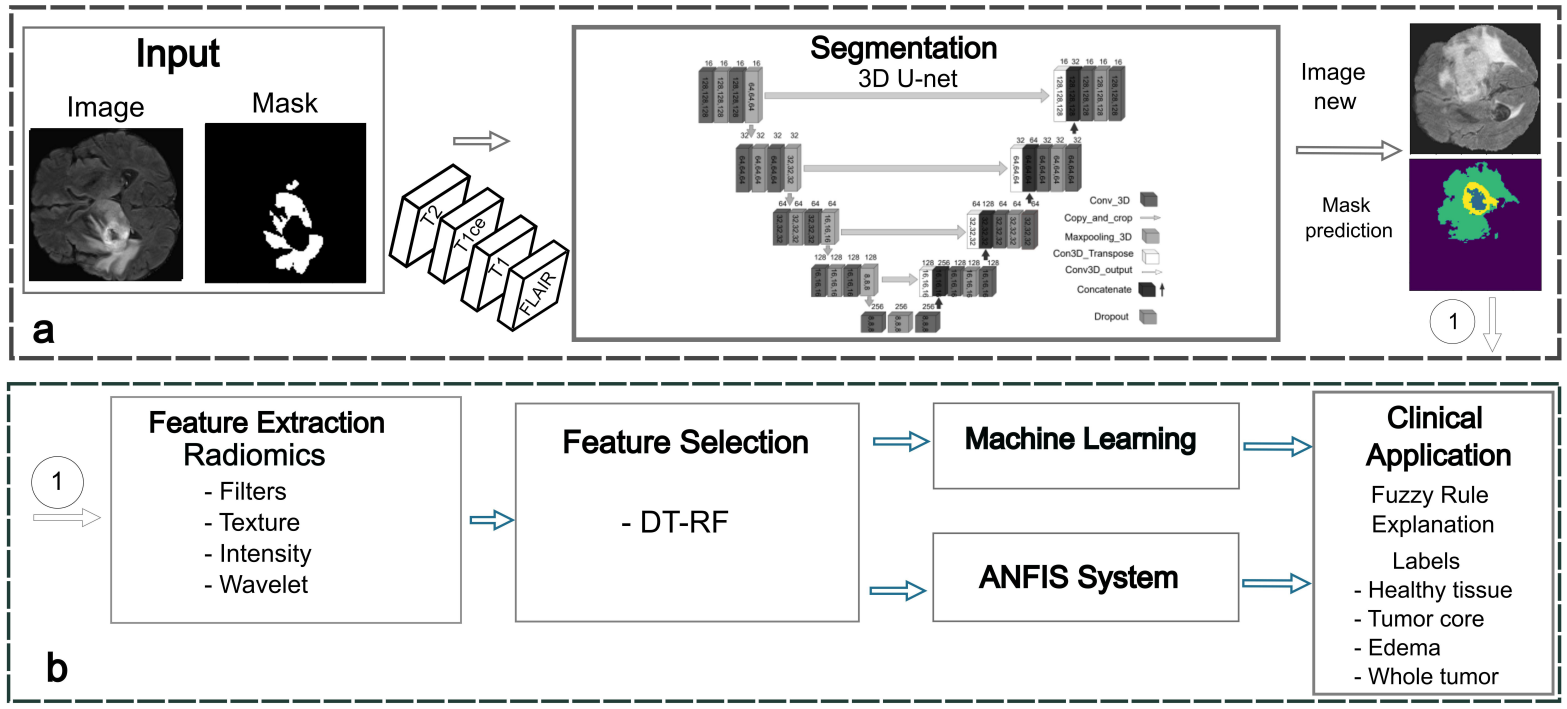
Proposed framework for BT detection. **(a)** Phase 1: segmentation stage for accurate delineation of tumor regions. **(b)** Phase 2: feature extraction and selection using radiomics. Phase 3: classification using machine learning and ANFIS techniques. Phase 4: fuzzy rules generation and optimization.

The proposed framework consists of four key stages: segmentation to identify regions of interest (ROI), feature extraction, feature selection, and classification using a fuzzy system to improve explainability (Figure 1). The following sections provide a detailed description of the methodology, highlighting each step in the process.

#### 3.2.1 Phase 1: tumor segmentation using deep learning

A U-Net-like Convolutional Neural Network (CNN), initially introduced by Basnet et al. (2021), was utilized for the segmentation phase. Although the network was originally designed to segment gray matter, white matter, and cerebrospinal fluid (CSF), it was later adapted to segment BT on multimodal MRI. The 3D U-Net architecture employed in this framework enables the precise delineation of tumor boundaries by accounting for variations in tumor geometry and adjacent cerebral structures. The model integrates T1-weighted, T1ce, T2-weighted, and FLAIR sequences, each providing complementary imaging characteristics of tumor regions. For instance, T1-weighted images are crucial for identifying pathological changes and delineating tumor contours. Areas of abnormal vascularity and blood-brain barrier (BBB) breakdown, commonly observed in malignant tumors, appear highlighted in T1-weighted images (Shiroishi et al., 2015; Paek et al., 2013). The T1ce sequence, enhanced with a gadolinium-based contrast agent, improves tumor visibility by emphasizing regions where the BBB is compromised, making it particularly valuable for tumor characterization. This distinction between tumor tissue and healthy brain parenchyma is essential for surgical planning and treatment decisions (Paek et al., 2014). In glioblastoma, for example, the degree of contrast enhancement correlates with tumor aggressiveness and BBB disruption, making T1ce a critical modality for assessing tumor burden and guiding surgical interventions (Hattingen et al., 2017). T2-weighted and FLAIR images play a key role in evaluating edema and necrosis, both critical for tumor characterization. T2-weighted images highlight hyperintense regions of vasogenic edema, often associated with BBB disruption (Champ et al., 2012; Hung et al., 2023). FLAIR sequences suppress cerebrospinal fluid signals, improving the visualization of cortical and periventricular lesions, thus aiding in the detection of infiltrative tumor components that may not be visible with contrast-enhanced imaging (Zúñiga et al., 2023). This is particularly important in gliomas, where neoplastic cells often extend beyond the regions of enhancement (Huse et al., 2013; Zeineldin et al., 2020). The 3D U-Net architecture processes volumetric data through 3D convolutions, max-pooling, and upsampling operations, capturing spatial dependencies and contextual information across adjacent MRI slices. This approach is fundamental for accurately segmenting tumor regions and distinguishing between various tumor subtypes with high precision. The modified architecture used patch-wise learning, where 128 × 128 × 128 patches were extracted from input images at each training step [similar to the proposal of Mellado et al. (2023)].

The loss function, which combines cross-entropy and DICE loss (Equation 1), was used to quantify performance at the end of each epoch. The Adaptive Moment Estimation (ADAM) optimizer was then applied to update the model parameters accordingly.


(1) $$\begin{array}{l} \begin{array}{l} L_{C}=L_{CE}+L_{DICE}\\ \;=-\frac{1}{N}\overset{N}{\underset{b=1}{\sum }}\left(\frac{1}{2}\overset{M}{\underset{c=1}{\sum }}Y_{b,c}\cdot \log\left({\hat{Y}}_{b,c}\right)+\overset{M}{\underset{c=1}{\sum }}\frac{2\cdot Y_{b,c}\cdot {\hat{Y}}_{b,c}}{Y_{b,c}+{\hat{Y}}_{b,c}}\right) \end{array} \end{array}$$

The loss function evaluates the probability that a prediction for the input image *b* belongs to label *c* for both the ground truth segmentation *Y* and the network-predicted segmentation Ŷ. Here, *N* represents the batch size, *b* is the image input sample, and *c* denotes the segmentation label. The network was trained for 300 epochs using a batch size of 8 and an initial learning rate of 2 × 10^−4^, which was halved every 30 epochs to improve convergence.

#### 3.2.2 Phase 2: feature extraction and selection using radiomics

**Figure 2:**
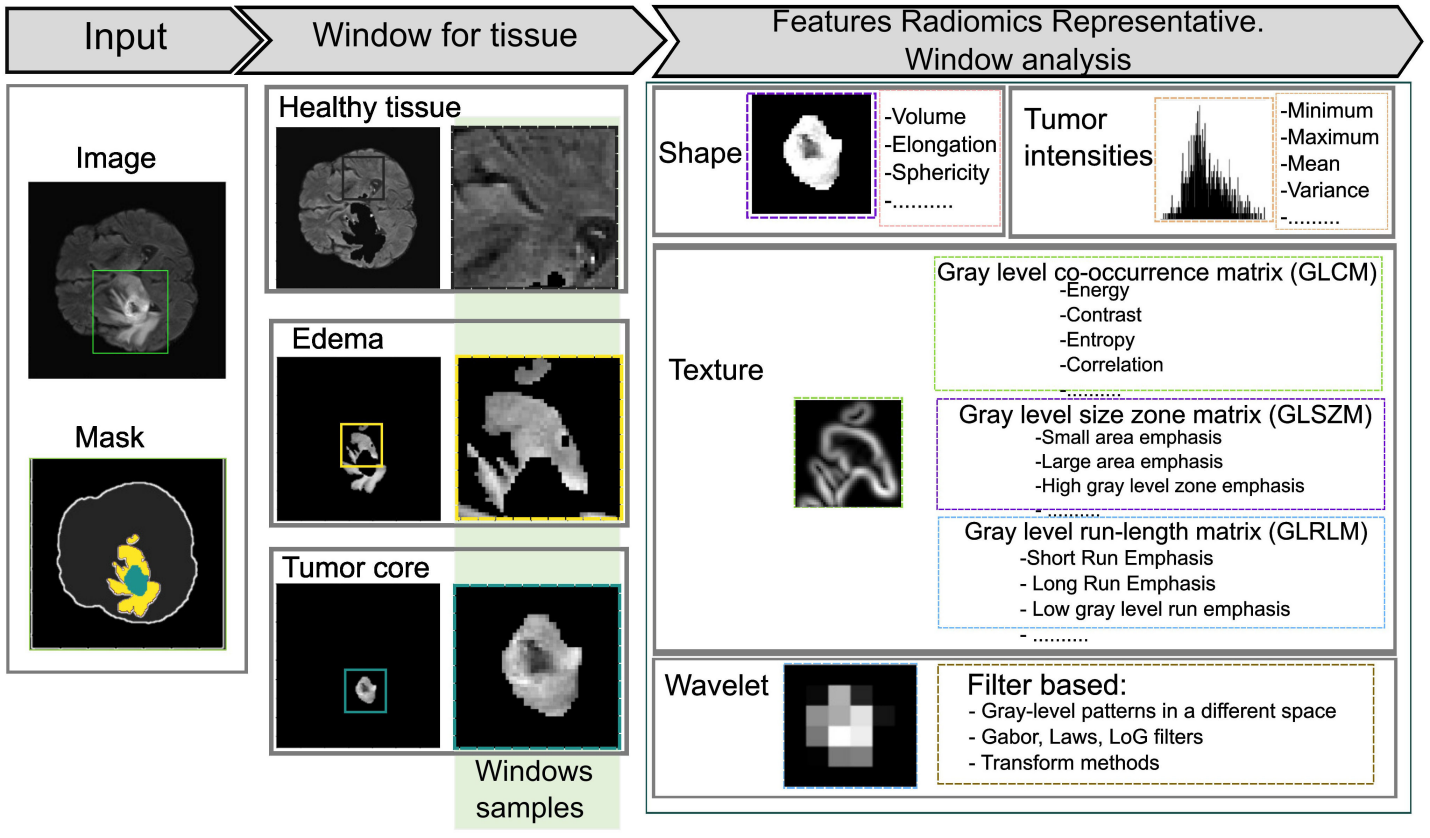
Representation of radiomic features using various statistical approaches. A 45 × 45 kernel is applied to extract features within a defined window of the region of interest (ROI).

Radiomics involves the extraction of a large number of quantitative features from medical images, generating variables that can be analyzed to support clinical decision-making and enhance diagnostic accuracy (Saini et al., 2023). By transforming medical images into high-dimensional data, radiomics enables the identification of complex patterns and correlations that provide a deeper understanding of the lesion or region of interest. The extracted features are typically classified into several types as shown in Figure 2.

- Morphological features: describe the shape and volume of anatomical structures or lesions, providing information on their geometric properties.
- Histogram-based (first-order) features: quantify the distribution of pixel or voxel intensities, reflecting overall intensity patterns within the region of interest (ROI).
- Texture-based (second-order) features: such as those derived from the Gray Level Co-occurrence Matrix (GLCM) and Gray Level Dependence Matrix (GLDM), capture spatial relationships and variability in gray levels, offering information about the complexity and heterogeneity of tissues.
- Transformation-based features: including wavelet or log-sigma transformations highlight details at multiple scales or frequencies, revealing finer structural details.

**Figure 3:**
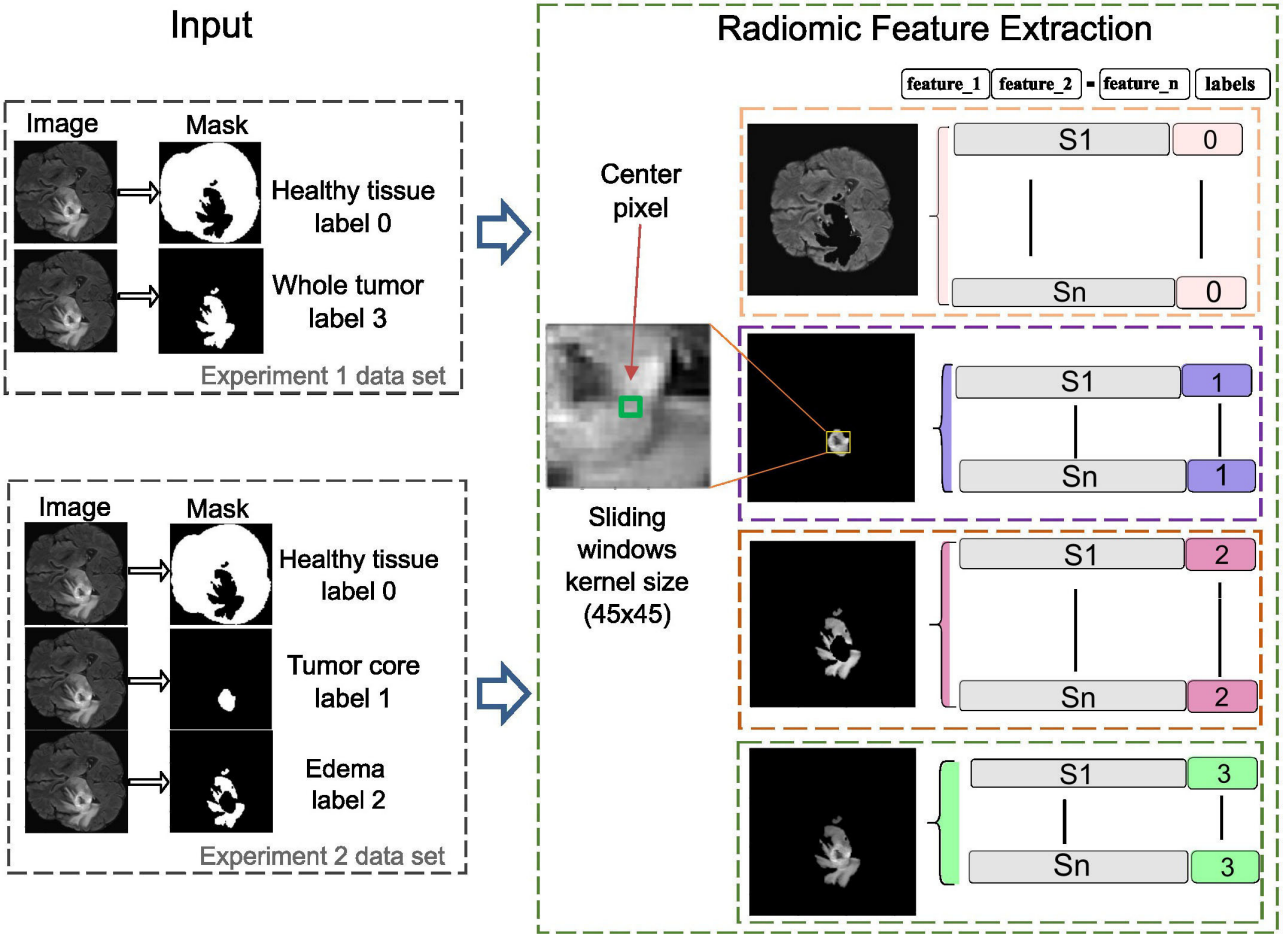
The radiomics-based feature extraction methodology employs sliding window analysis to construct a feature matrix, with columns representing extracted features and rows corresponding to labeled objects in the images. A 45 × 45 kernel is used to enhance detail in each image segment, improving the accuracy of clinically relevant diagnoses.

In this phase, radiomic features are extracted using sliding window analysis with a 45 × 45 kernel to capture fine details between different ROI about healthy tissue, whole tumor, tumor core, and edema according to the experiments carried out on the study image. Each window is processed using the SimpleITK interface to generate images and masks compatible with feature extraction. PyRadiomics toolbox is used to perform a quantitative analysis of radiomic features for each region while preserving its association with the original image and its corresponding label. The resulting feature matrix is constructed by organizing the computed characteristics into columns, while the objects associated with each label are arranged in rows, as illustrated in Figure 3. This method increases the data dimensionality because of the large number of calculated parameters.

Feature selection is a critical step in radiomics analysis, particularly in medical imaging, where extracted features often include irrelevant or redundant information, which can negatively impact model performance. Various techniques, including Sequential Forward Selection (SFS), Sequential Backward Selection (SBS), Recursive Feature Elimination (RFE), and Least Absolute Shrinkage and Selection Operator (LASSO), are widely employed to identify the most relevant features (Naveed et al., 2021; Johnpeter and Ponnuchamy, 2019; Bhattacharjee et al., 2022; Zhang J. et al., 2022). Among feature selection approaches, Decision Trees (DT) effectively assess feature importance, reduce dimensionality, and enhance classification accuracy (Kutikuppala et al., 2023; Paja, 2016). Similarly, Random Forest (RF) is well-suited for handling high-dimensional data and provides robust feature importance rankings (Lefkovits et al., 2017; Kumar et al., 2023).

#### 3.2.3 Phase 3: classification using machine learning and ANFIS models

In this phase, several conventional machine learning models were implemented to assess their performance in BT classification tasks using the radiomic features extracted during the previous phase. These models include:

- Linear Discriminant Analysis (LDA): a statistical method that projects data onto a lower-dimensional space to maximize class separability by modeling the relationship between input features and class labels through linear decision boundaries.
- Decision Trees (DT): a rule-based model that partitions the dataset into subsets based on feature values, creating a hierarchical tree structure where each internal node represents a decision rule and the leaf nodes correspond to predicted outcomes.
- Random Forest (RF): an ensemble learning technique that combines multiple decision trees to improve accuracy and robustness. Reduce overfitting by averaging predictions from various trees, making it particularly effective for complex datasets.
- Logistic Regression (LR): a probabilistic model used for binary or multi-class classification that estimates the likelihood of class membership based on a logistic function applied to weighted input features.
- Multilayer Perceptron (MLP): a type of artificial neural network consisting of multiple layers of interconnected neurons. It learns complex, non-linear relationships in the data through backpropagation and is well-suited for high-dimensional feature spaces.
- k-Nearest Neighbors (k-NN): a non-parametric, instance-based learning algorithm that classifies data points by comparing them to their nearest neighbors in the feature space, relying on majority voting to determine class membership.

The Adaptive Neuro-Fuzzy Inference System (ANFIS) model, introduced by Jang (1993), combines neural networks with fuzzy logic systems to form a neuro-fuzzy framework. Built on the Takagi-Sugeno (TS) fuzzy inference system, ANFIS utilizes fuzzy logic for rule-based decision-making while adapting its parameters based on input data (Anggara and Munandar, 2023; Allende-Cid et al., 2016; Querales et al., 2023).

**Figure 4:**
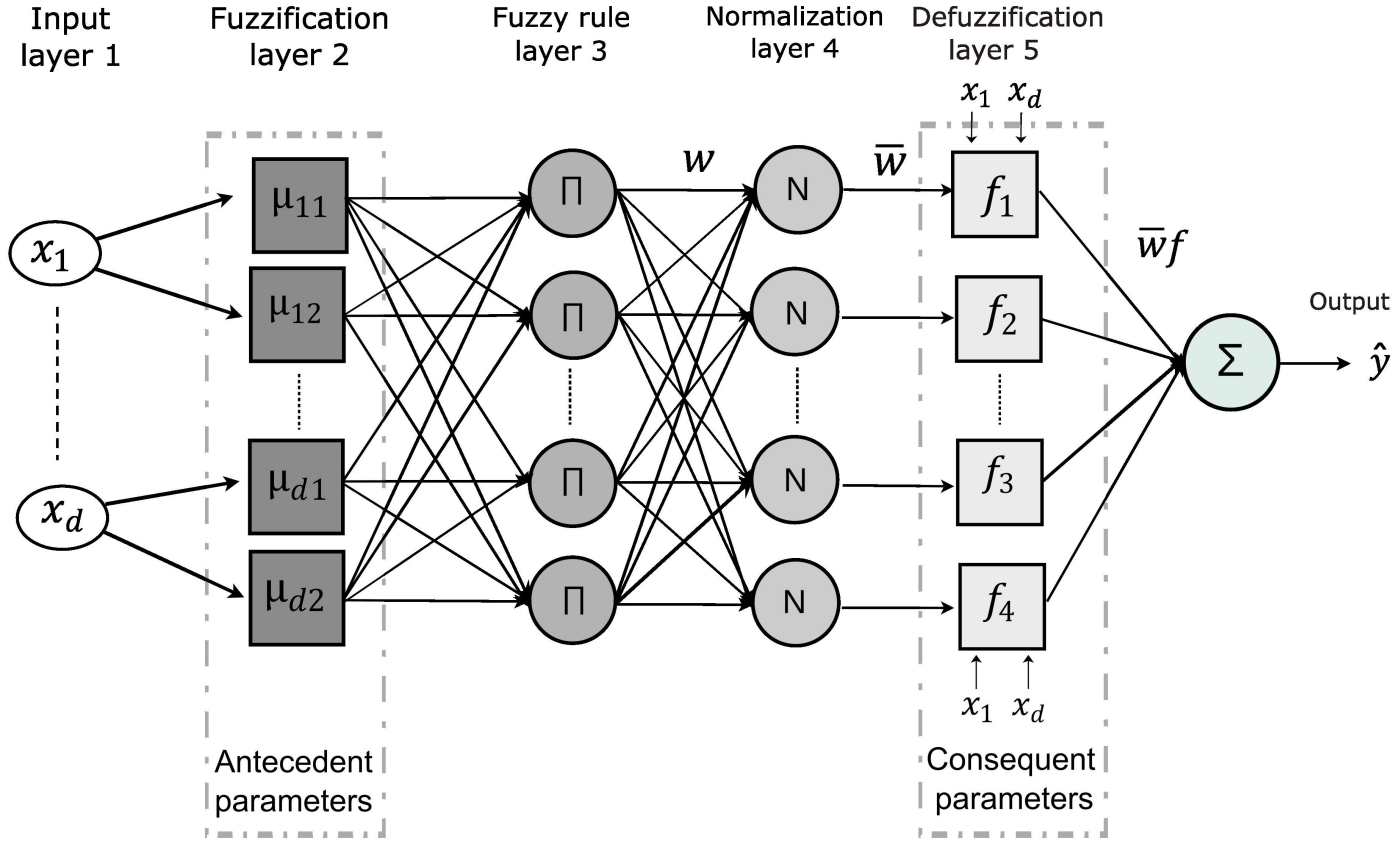
The ANFIS model architecture highlights the key parameters involved in decision rule development. The second layer performs fuzzification, defining membership functions for each input variable and extracting antecedent parameters. In the fifth layer, defuzzification computes the consequent parameters. The classification process culminates in the generation of decision rules and the interpretation of the model's outputs.

The architecture of ANFIS, illustrated in Figure 4, consists of multiple layers dedicated to processing fuzzy rules. The input layer applies Gaussian membership functions to perform the fuzzification of the input data. These membership functions are mathematically defined as follows:


(2) $$\begin{array}{l} \mu_{kj}\left(x_{j}\right)=\exp\left[-\left(\frac{{\left(x_{j}-m_{kj}\right)}^{2}}{\sigma_{kj}^{2}}\right)\right], \end{array}$$

where *m*_*kj*_ denotes the mean and σ_*kj*_ the standard deviation, determining the center and width of each Gaussian function. After the inputs are fuzzified, the model calculates the firing strength of each fuzzy rule. This is done by computing the product of the membership values for all input variables associated with the corresponding rule:


(3) $$\begin{array}{l} w_{k}\left(\vec{x}\right)=\overset{n}{\underset{j=1}{\prod }}\mu_{kj}\left(x_{j}\right), \end{array}$$

where the product aggregates the degrees of membership, indicating the extent to which the inputs satisfy each rule's antecedents. The firing strengths are then normalized by dividing each rule's strength by the sum of all rule strengths. This ensures that the relative contribution of each rule is proportionate:


(4) $$\begin{array}{l} {\bar{w}}_{i}=\frac{w_{i}}{\sum_{j=1}^{R}w_{j}}. \end{array}$$

This normalization step ensures that the combined influence of the rules remains balanced and consistent across the system. The normalized firing strengths are then applied to the linear functions representing the fuzzified inputs. The output of each rule is weighted according to its firing strength, contributing proportionally to the final prediction. The overall output of the model is computed by summing the weighted contributions from all rules:


(5) $$\begin{array}{l} \hat{y}=\overset{K}{\underset{i=1}{\sum }}{\bar{w}}_{i}f_{i}=\overset{K}{\underset{i=1}{\sum }}{\bar{w}}_{i}\left(\overset{K}{\underset{j=1}{\sum }}p_{ij}x_{j}+q_{i}\right), \end{array}$$

Here, *p*_*ij*_ and *q*_*i*_ represent the consequent parameters. This final equation computes the system's output by integrating the contributions of all the rules applied to the input data, yielding the final prediction or classification.

#### 3.2.4 Phase 4: fuzzy rule generation and optimization

The main functionality of the ANFIS model is the *fuzzy IF-THEN rules*, which provide a transparent and interpretable way of mapping input variables to output decisions. In ANFIS, the antecedents of these rules are represented by fuzzy sets that describe the degree to which input variables satisfy certain conditions, while the consequents are typically expressed as linear functions or singletons representing the system's output classes.

These rules follow the Takagi-Sugeno formulation, where the premise of each rule maps the input vectors to the corresponding output functions *y*_*k*_ = *f*_*k*_(*x*). Each rule evaluates a specific condition and contributes proportionally to the final output. Mathematically, this can be expressed as:


(6) $$\begin{array}{l} \text{Rule k: IF }x_{1}\text{is }\mu_{k1}\text{ AND }x_{2}\text{ is }\mu_{k2}\text{ AND }...\text{ AND }x_{n}\text{ is }\mu_{kd}\\ \text{ THEN }y_{k}=f_{k}\left(x\right),\text{ for }k=1,2,\ldots ,K \end{array}$$

Here, μ_*kd*_ represents a fuzzy set associated with the input variable *x*_*d*_ for the *k*-th rule, *K* denotes the total number of fuzzy rules and *Y* is a fuzzy aggregation operator used to combine the outputs of all rules. It employs fuzzy *IF-THEN* rules, where the antecedents are fuzzy sets associated with input variables, and the consequents are fuzzy singletons representing output classes.

On the other hand, the Particle Swarm Optimization (PSO) algorithm was utilized to optimize the rules within the ANFIS model (Shihabudheen et al., 2018). Inspired by the collective behavior of animals such as fish or birds, the PSO adjusts both the antecedents and the consequences of the decision rules by treating the data as a swarm of particles (Shami et al., 2022). In the *PSO-ANFIS* framework, ANFIS serves as a particle, while PSO complements it by fine-tuning its parameters to identify the most optimal solution (Moayedi et al., 2020). Each particle is characterized by two key attributes: velocity estimation and position update, which are iteratively optimized during the learning process.

In an *n*-dimensional space, the position of a particle, *x*_*i*_(*t*), is updated by adding its velocity, *v*_*i*_(*t*), to the current position:


(7) $$\begin{array}{l} x_{i}\left(t+1\right)=x_{i}\left(t\right)+v_{i}\left(t\right) \end{array}$$

The velocity is calculated as:


(8) $$\begin{array}{l} v_{i}\left(t\right)=c_{1}r_{1}\left(pbest\left(t\right)-x_{i}\left(t\right)\right)+c_{2}r_{2}\left(gbest\left(t\right)-r_{i}\left(t\right)\right) \end{array}$$

where *c*_1_ and *c*_2_ are acceleration coefficients, *r*_1_ and *r*_2_ are random vectors, and *pbest* and *gbest* represent the best local position of the particle and the best global position, respectively.

For classification tasks, the *PSO-ANFIS* model relies on initial parameter settings derived from the literature review (Mercangöz, 2021; Gad, 2022; Shami et al., 2022). The process begins with the preparation of the data set, including collection, normalization, and preprocessing. Parameters such as particle population size, acceleration coefficients, number of iterations, and linguistic fuzzy sets are then configured. The initial adjustment of the model is crucial to obtain a good performance. For this reason, the bibliographies consulted (Adewuyi et al., 2022; Shami et al., 2022; Mercangöz, 2021), help to choose the group of informants equal to 15, the confidence coefficient corresponding to 2.05, the velocity factor of 0.9, the number of agents equal to 40, the number of iterations equal to 200, the variation in the mean of the premise functions corresponding to 0.2, the center premise functions and standard deviation corresponding to 0.5 and 0.2 respectively, the exponent range of the premise function between 1.0 and 3.0 and finally the range of values for the consequent functions between -10.0 and 10.0.

To better understand the proposed approach, the pseudocode in Algorithm 1 outlines how *PSO-ANFIS* is employed to solve classification problems for regions of interest (ROIs). The process begins by setting the PSO parameters, including the number of particles (*nPop*), the number of iterations (*epochs*), the confidence coefficient (*phi*), and the velocity factor (*vel_fact*). The positions and velocities of the particles are initialized to represent the ANFIS parameters. During each iteration, the velocities and positions of the particles are updated based on cognitive components (each particle's best position) and social components (best global position swarm's). The performance of each configuration is evaluated using a cost function and the best positions and associated costs are recorded to guide the global search of the swarm. At the end of the process, the algorithm outputs the optimal configuration of the ANFIS parameter and the corresponding minimized classification error. Additionally, Algorithm 2 details the velocity update mechanism for each particle, which combines cognitive and social components to refine parameter adjustments. This iterative approach efficiently tunes the antecedent and consequent parameters of the ANFIS, significantly enhancing its classification performance.

**Algorithm 1:**
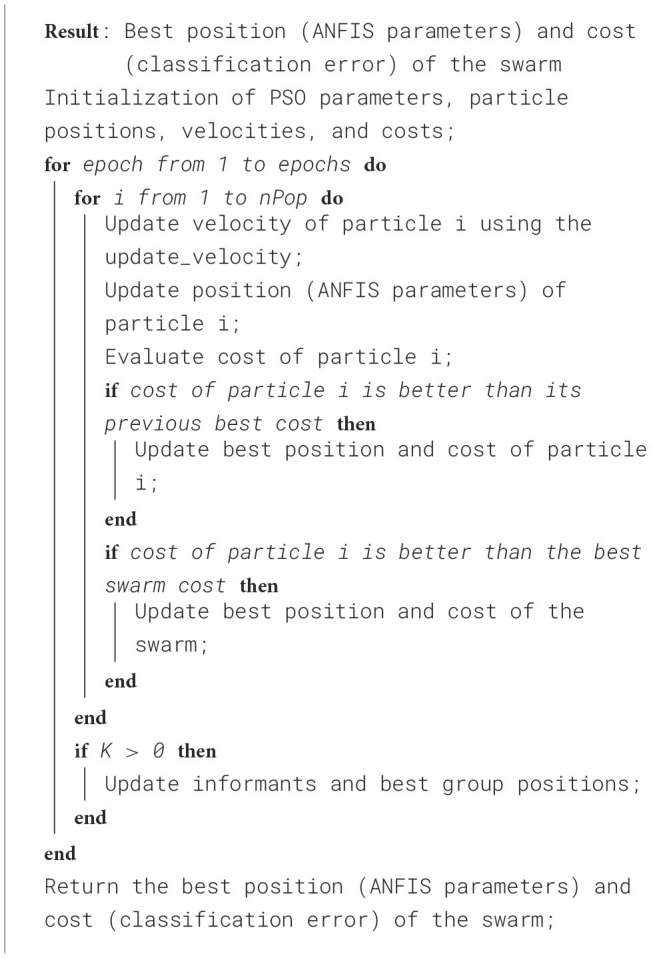
PSO_ANFIS_Classification.

**Algorithm 2:**
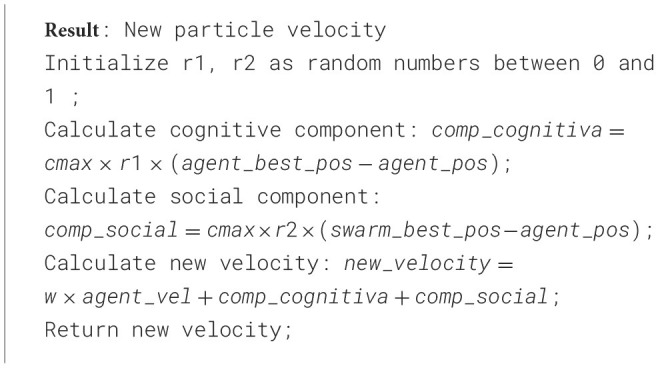
Update_velocity.

### 3.3 Performance metrics

The predicted labels from the test results were compared with the actual labels to construct a confusion matrix. Performance metrics such as accuracy, precision and F1-Score were calculated using the components of the confusion matrix: true positives (*T*_*P*_), false positives (*F*_*P*_), true negatives (*T*_*N*_) and false negatives (*F*_*N*_). Here, *T*_*P*_ represents the number of ROIs correctly classified as tumor, *F*_*N*_ denotes the number of tumor ROIs incorrectly predicted as normal, and *T*_*N*_ indicates the number of ROIs correctly classified as normal.

Performance metrics considered in this study were defined as follows:

- Accuracy: The proportion of correctly classified ROIs to the total number of ROIs:
  (9) $$\begin{array}{l} \text{Accuracy}=\frac{T_{P}+T_{N}}{T_{P}+T_{N}+F_{P}+F_{N}} \end{array}$$
- Precision: The proportion of correctly predicted tumor ROIs to all predicted tumor ROIs:
  (10) $$\begin{array}{l} \text{Precision}=\frac{T_{P}}{T_{P}+F_{P}} \end{array}$$
- Recall (Sensitivity): The proportion of correctly predicted tumor ROIs to all actual tumor ROIs:
  (11) $$\begin{array}{l} \text{Recall}=\frac{T_{P}}{T_{P}+F_{N}} \end{array}$$
- F1 Score: The harmonic mean of Precision and Recall, providing a balanced evaluation:
  (12) $$\begin{array}{l} \text{F1 Score}=\frac{2\cdot \text{Precision}\cdot \text{Recall}}{\text{Precision}+\text{Recall}} \end{array}$$

The DICE Score is a widely used metric for evaluating the overlap between two regions of interest (ROIs) in an image, particularly for segmentation tasks. It is defined as:


(13) $$\begin{array}{l} \text{DICE Score}=\frac{2\cdot |A\cap B|}{\left|A|+|B\right|} \end{array}$$

where *A* represents the predicted ROI, *B* represents the ground truth ROI, |*A* ∩ *B*| is the number of pixels (or voxels) common to both *A* and *B* (the intersection), |*A*| is the total number of pixels in the predicted ROI, and |*B*| is the total number of pixels in the ground truth ROI. A DICE Score close to 1 signifies a near-perfect overlap between the predicted and ground truth ROIs, whereas a score of 0 indicates no overlap at all.

## 4 Results

### 4.1 Segmentation results

The neural network was trained over 300 epochs, achieving an average DICE Score of 86.07% during the segmentation process. Convergence occurred around epoch 50, with minimal fluctuations in validation accuracy and the loss function approaching its minimum values. The test results showed as average and standard deviation of the DICE Score for segmentation of 82.94% ± 16.92 for tumor core (TC), 76.06% ± 17.27 for edema (E), and 99.90% ± 0.06 for healthy tissue.

**Figure 5:**
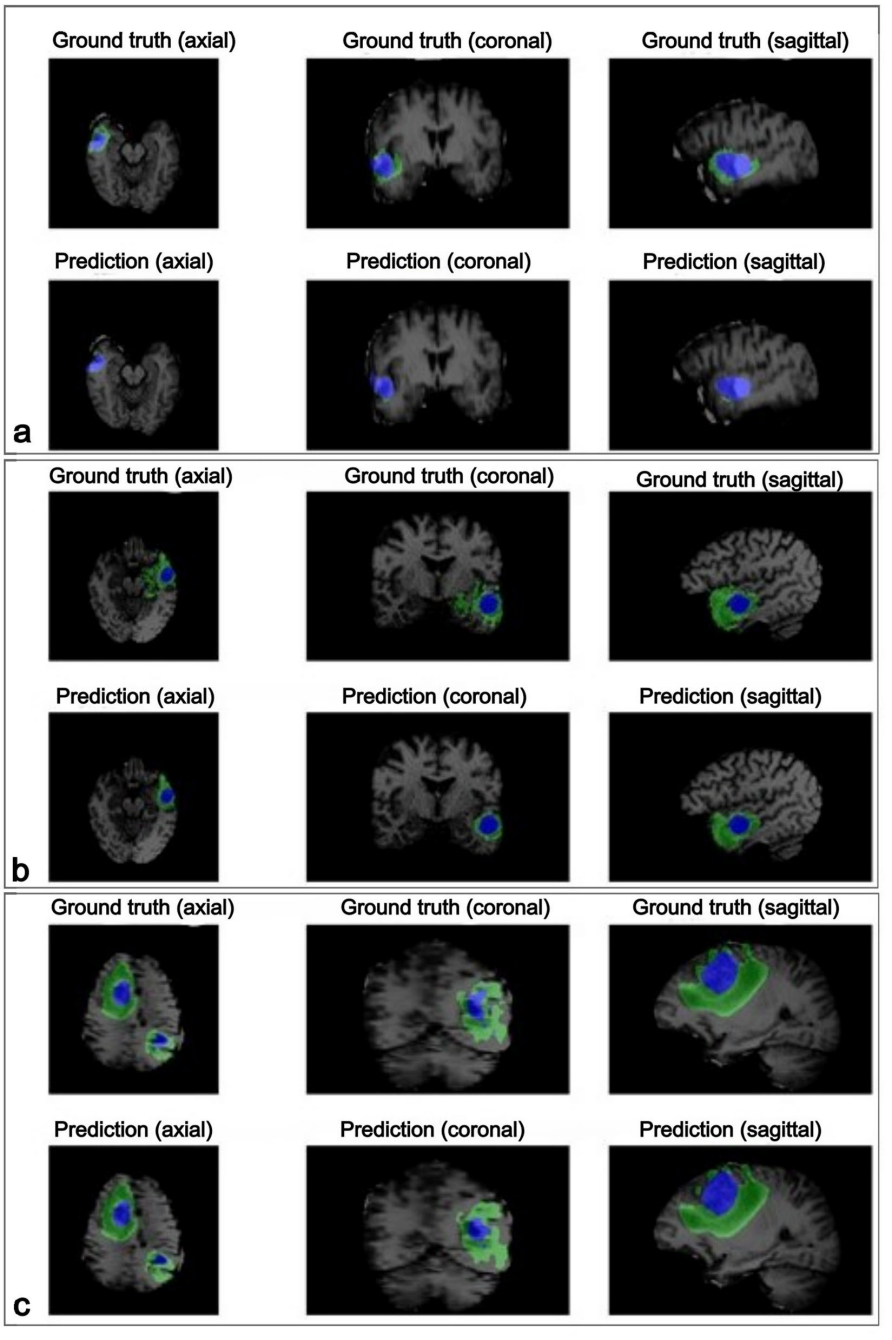
The visualization displays the ground truth and predicted tumor segmentation across axial, coronal, and sagittal planes for three representative subjects **(a–c)**. Each row presents the ground truth (top) and the model's prediction (bottom) for the same subject, highlighting the high similarity between the predicted and actual labeled tumor regions. The blue regions represent the tumor core, while the green regions indicate the edema surrounding the tumor core. Case **(a)** shows a small, localized tumor core with minimal edema; case **(b)** shows a moderately sized tumor core with more noticeable surrounding edema; and case **(c)** presents a large tumor with an extensive tumor core and widespread edema affecting multiple brain areas of the brain.

Further evaluation in three test subjects, visualized in Figure 5, illustrates the performance of the model in the axial, coronal, and sagittal planes, highlighting the predicted regions of interest (tumor core (TC), edema (E) and healthy tissue). For subject Figure 5a, the model achieved precisions of 90.47% for tumor core, 62.29% for edema, and 99.91% for healthy tissue. Subject Figure 5b exhibited precisions of 85.83% for the tumor core, 10.43% for edema, and 99.93% for healthy tissue. Lastly, for the subject Figure 5c, the model achieved a precision of 96.10% for the tumor core, 93.12% for edema, and 99.89% for healthy tissue.

These results highlight the model's ability to accurately segment healthy tissue and tumor core, with consistent performance across these classes. However, edema segmentation exhibited greater variability, highlighting it as a challenging aspect and underscoring the need for further refinement in this area.

### 4.2 Radiomic features selected by importance

After extensively evaluating various feature selection methods, a combination of the top-ranked features from Decision Trees (DT) and Random Forest (RF) yielded the best performance in our study. Features were ranked based on their importance scores, and the most relevant ones from both models were selected to identify the most discriminative attributes for label identification (Renugadevi et al., 2023; Srinivasan et al., 2019). To ensure consistency and prevent numerical scale differences from impacting model performance, all features were normalized to a range of 0 to 1. Among the 218 extracted features, the top 20 were selected for further analysis based on their importance scores, ensuring that the most informative attributes were retained for classification.

**Figure 6:**
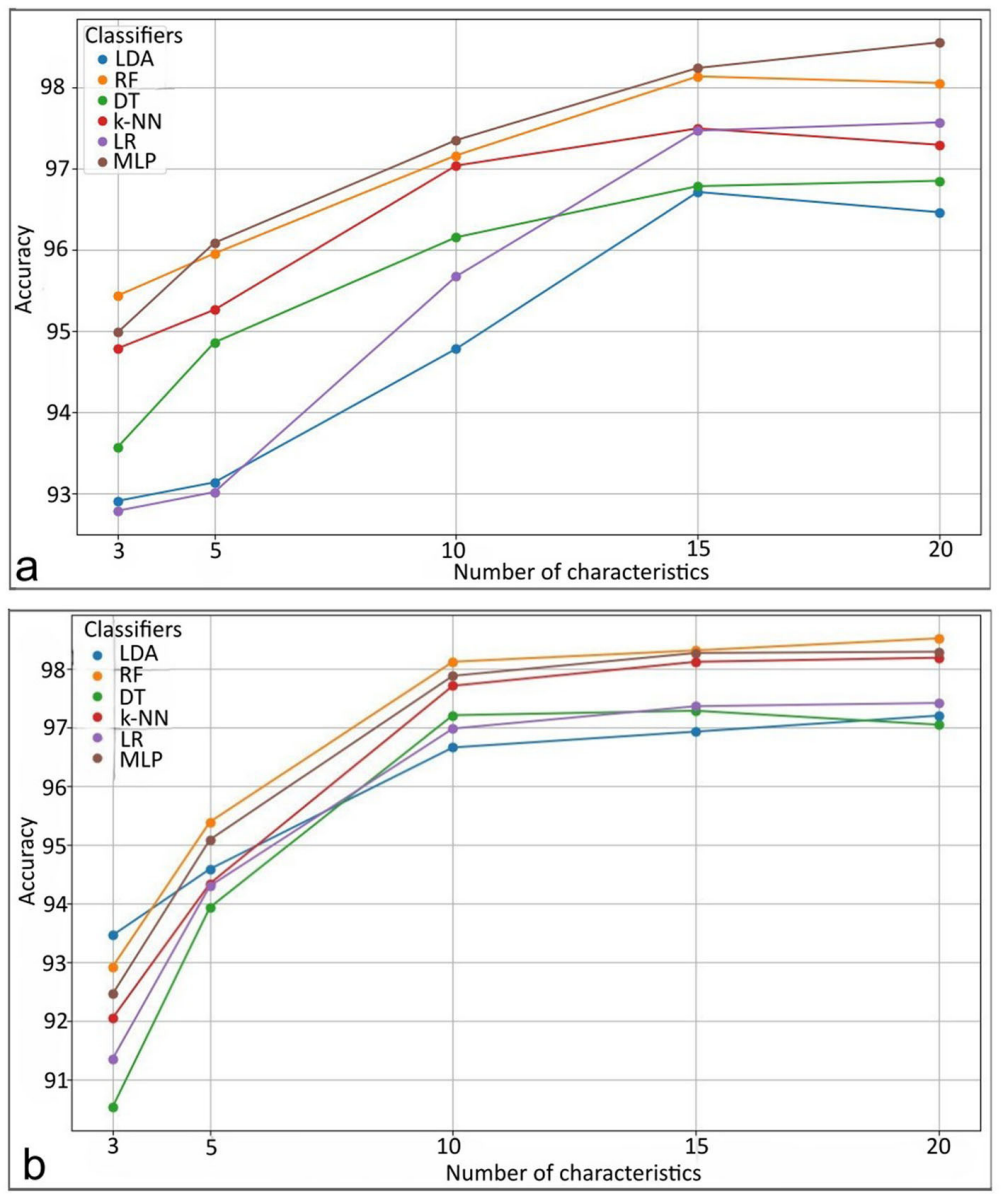
Performance results of the classifiers. For **(a)** distinguishing between healthy and affected tissue. For **(b)** distinguishing between healthy tissue, tumor core, and edema. LDA, Linear Discriminant Analysis; RF, Random Forest; DT, Decision Trees; k-NN, k-Nearest Neighbors; LR, Logistic Regression; MLP, Multilayer Perceptron.

An exploratory analysis was conducted to assess the performance of various machine learning (ML) models for BT classification, varying the performance according to the number of features used. As illustrated in Figure 6, model performance improved with increasing features in both experiments. Among the models, the Multilayer Perceptron (MLP) demonstrated the highest performance in distinguishing between healthy and affected tissue. The Random Forest (RF) model performed closely, showing strong accuracy in classifying healthy tissue, tumor core (TC), and edema (E). In contrast, a minimal set of three features was also tested, but yielded the lowest performance compared to larger feature sets.

Table 2 lists the top 10 most influential features of both experiments, ranked by relevance according to DT-RF methods. These findings suggest that performance comparable to existing proposals can be achieved using a subset of up to three features. For rule-based models such as ANFIS, this approach facilitates interpretability by using a smaller number of selected features, providing more precise explanations without sacrificing classification performance.

**Table 2:** Top 10 features for classification problems with their respective importances.

**Feature ID**	**Feature name**	**DT importance**	**RF importance**
**Binary-class problem: healthy tissue vs. whole tumor**			
10	log-sigma_glcm_ClusterShape	0.6234	0.1261
41	wavelet-L_gldm_GrayLevelNonUniformity	0.1358	0.1094
0	original_firstorder_Energy	0.0384	0.0577
32	log-sigma_glcm_JointEnergy	0.0302	0.0361
2	original_firstorder_Kurtosis	0.0184	0.0294
3	original_firstorder_Maximum	0.1044	0.1354
7	original_firstorder_Skewness	0.0122	0.0140
42	wavelet-L_gldm_HighGrayLevelEmphasis	0.0114	0.0061
35	wavelet-H_gldm_GrayLevelNonUniformity	0.0108	0.0662
8	original_firstorder_Variance	0.0103	0.0148
**Multi-class problem: healthy tissue vs. tumor core vs. edema**			
85	log-sigma_glcm_ClusterShape	0.2920	0.0329
1	original_shape_MaximumDiameter	0.2862	0.0444
91	log-sigma_glcm_JointEnergy	0.0384	0.0577
0	original_shape_Perimeter	0.0510	0.0301
2	original_shape_Elongation	0.0412	0.0094
70	log-sigma_gldm_HighGrayLevelEmphasis	0.0392	0.0255
128	original_firstorder_Skewness	0.0122	0.0140
42	log-sigma_firstorder_Maximum	0.0126	0.0128
134	log-sigma_glrlm_HighGrayLevelRunEmphasis	0.0125	0.0107
97	log-sigma_glrlm_LowGrayLevelRunEmphasis	0.0119	0.0116

Several studies have identified key radiomic features that enhance the predictive accuracy of models in BT classification. Zhang et al. (2020) reported that specific textural characteristics, such as cellularity and peritumoral edema, vary between tumor types and play a significant role in classification. Çinarer et al. (2020) highlighted the importance of wavelet-based radiomic features in predicting glioma grades, demonstrating a strong association between certain wavelet features and tumor grade as well as patient survival outcomes. Similarly, Choi et al. (2020) emphasized the role of Gray Level Co-occurrence Matrix (GLCM) features in quantifying glioblastoma texture patterns, noting that contrast, correlation, energy, and homogeneity serve as strong prognostic factors. Chen et al. (2018) used a diverse set of radiomic features–including 18 first-order features, 13 shape features, and 74 texture features–to effectively identify gliomas. The findings from these studies align with the current work, which highlights radiomic features that capture variability in gray level, texture, complexity, and contrast through advanced transformation techniques. Key feature categories include first-order statistics, GLCM, Gray Level Dependence Matrix (GLDM), and shape features. Additionally, intensity analysis, energy, and clustering metrics are extracted from multiple image domains, including original, log-sigma, and wavelet-transformed images, providing a comprehensive representation of tumor characteristics.

### 4.3 Evaluation of the classifier performances

The use of fewer features improves the interpretability of the classification results, facilitating clearer insights into the decision-making process. The performance results for two experiments are summarized in Tables 3, 4. The study used 5-fold cross-validation to evaluate various methodologies for tissue classification. The dataset was systematically partitioned into 80% training and 20% validation data for each of the 5 folds, subsequently quantifying the mean and standard deviation of the classification accuracies in the 5 folds within the validation samples. Afterwards, the test set comprising fully independent data demonstrates the generalization performance.

**Table 3:** Validation and test results for the binary classification: *healthy* tissue vs. *whole tumor*.

**Machine learning model**	**Validation**	**Test**						**Accuracy**
	**Accuracy std**	**Precision**		**Recall**		**F1-score**		
		**Healthy**	**Tumor**	**Healthy**	**Tumor**	**Healthy**	**Tumor**	
LDA	88.62 ± 7.83	95.92	74.44	95.83	74.86	95.87	74.65	92.90
RF	92.76 ± 4.75	97.99	81.08	96.68	87.76	97.33	84.29	95.43
DT	91.06 ± 4.42	97.54	73.12	94.92	85.23	96.21	78.71	93.57
k-NN	92.51 ± 4.55	97.94	77.84	95.96	87.53	96.94	82.40	94.78
LR	90.81 ± 6.05	97.72	69.35	93.80	86.51	95.72	76.99	92.78
MLP	93.65 ± 3.81	98.32	77.67	95.81	89.88	97.05	83.33	94.98
ANFIS	94.06 ± 0.25	98.38	78.12	96.35	90.24	97.32	84.20	95.20

**Table 4:** Validation and test results for the multi-class problem: *healthy* tissue (HT) vs. *tumor core* (TC) vs. *edema* (E).

**Machine learning model**	**Validation**	**Test**									**Accuracy**
	**Accuracy std**	**Precision**			**Recall**			**F1-score**			
		**HT**	**TC**	**E**	**HT**	**TC**	**E**	**HT**	**TC**	**E**	
LDA	85.55 ± 3.49	97.71	58.52	88.76	96.07	72.73	88.76	96.89	64.85	85.81	92.47
RF	85.62 ± 3.96	98.12	60.09	74.19	95.30	70.19	74.19	96.69	64.75	80.58	92.93
DT	82.38 ± 3.82	97.90	51.28	62.88	93.32	61.17	62.88	95.55	55.79	73.43	90.55
k-NN	85.28 ± 3.76	98.03	55.57	72.72	94.43	69.55	72.72	96.20	61.78	79.19	92.06
LR	86.27 ± 3.03	98.03	50.52	78.09	93.46	74.05	78.09	95.69	60.06	80.79	91.36
MLP	87.26 ± 2.63	98.51	55.17	80.00	94.26	80.76	80.00	96.34	65.56	81.11	92.47
ANFIS	89.11 ± 0.31	99.10	59.24	65.52	93.44	80.82	87.67	96.18	68.36	74.99	92.14

In Experiment 1 (Table 3), which focused on distinguishing healthy tissue from tumor tissue, the Random Forest (RF) and ANFIS classifiers outperformed other models, achieving greater accuracy than 95% in the test data. This high accuracy underscores their strong detection capabilities. ANFIS demonstrated superior performance, with higher accuracy and lower variability during cross-validation, exhibiting a standard deviation of ±0.25%. This stability highlights its robustness and consistency across different data splits, reinforcing its reliability. Furthermore, both RF and ANFIS recorded the highest F1-score for individual labels, surpassing 97% for healthy tissue and 84% for tumor tissue.

In Experiment 2, which involved multiclass classification, several methods, including LDA, RF, k-NN, MLP, and ANFIS, achieved excellent results, with test accuracies exceeding 92%, demonstrating their efficacy in handling more complex medical image classification tasks. ANFIS obtained the lowest variability among all models (standard deviation = ±0.31), further strengthening its reliability. LDA excelled in classifying tissue of edema, achieving an F1-score of 85.81%, while ANFIS showed strength in identifying the tumor core, with an F1-score exceeding 68%. This highlights the ability of ANFIS to combine interpretability while preserving good performance using its rule-based approach.

### 4.4 Interpretability analysis

**Figure 7:**
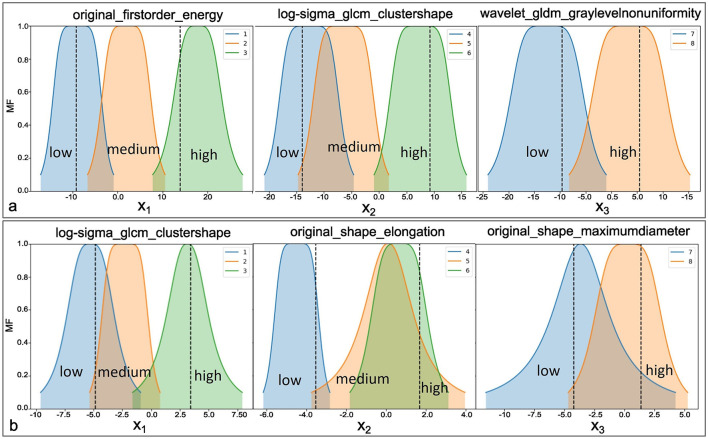
Representation of the membership functions generated by the ANFIS model. For **(a)** labels *healthy tissue* vs. *whole tumor*, and **(b)** for labels *healthy tissue* vs. *tumor core* vs. *edema*, divided into fuzzy subsets for both experiments.

The three most relevant radiomics features, selected according to their importance, as shown in Table 2, were used as input for the ANFIS model. Each feature was assigned linguistic variables according to its value ranges, following a structure of eight variables (3,3,2), where the first two features had three linguistic terms and the third had two. The membership functions for the three selected radiomic features are shown in Figure 7a for the analysis of healthy tissue and whole tumor, Figure 7b for the analysis of healthy tissue, tumor core and edema.

The selected radiomic features are modeled using linguistic variables in the second experiment. Similarly to experiment 1, the first two features are divided into three terms: “low,” “medium,” and “high,” while the third feature is divided into two terms: “low” and “high.” However, in this case, the analysis focuses on three tissue classes: healthy tissue, tumor core, and edema. The selection of these features, based on their quantitative importance, underscores the need for expert knowledge to ensure that the variables selected represent meaningful patterns in the classification of tissue types. These linguistic variables enable the creation of fuzzy rules that capture the nonlinear relationships between input features and tissue classes, providing accurate classification and interpretability.

In both experiments, the selected features help interpret the tissue portions (healthy tissue, tumor core, edema, and whole tumor) listed below in Table 5.

**Table 5:** Feature selection for Experiment 1 and Experiment 2.

**Order**	**Feature category**	**Feature names**	**Image type**
**Experiment 1: binary classification**, ***Healthy tissue*** **vs**. ***whole tumor***			
1	firstorder	Energy	original
2	glcm	ClusterShape	log-sigma
3	gldm	GrayLevelNonUniformity	wavelet
**Experiment 2: multiclass classification**, ***Healthy tissue*** **vs**. ***tumor core*** **vs**. ***edema***			
1	glcm	ClusterShape	log-sigma
2	shape	Elongation	original
3	shape	MaximumDiameter	original

Expert knowledge was essential in defining the appropriate ranges for linguistic variables to analyze the selected radiomic features. The membership functions corresponding to these features are illustrated in Figure 7a for experiment 1 and Figure 7b for experiment 2. Furthermore, the structure of the linguistic variables and the number of fuzzy rules remained consistent between both experiments, ensuring comparable conditions for the classification tasks. The Cartesian product of these variables generated 18 fuzzy rules that combine antecedents (based on the features) with consequences (the system outputs). These rules capture the non-linear relationships between inputs and tissue labels, allowing the interpretation of the results, as shown in Tables 6, 7.

**Table 6:** ANFIS rules: antecedents and consequents for labels *healthy tissue* vs. *whole tumor*.

**Rules**	**Antecedent**			**Consequent output**	
	*x*_1_	*x*_2_	*x*_3_	**Healthy tissue**	**Whole tumor**
1	l	l	l	1.40 − 3.79*x*_1_ − 0.30*x*_2_ − 3.52*x*_3_	− 5.56 − 4.73*x*_1_ − 0.64*x*_2_ + 0.88*x*_3_
2	l	m	l	7.32 + 1.22*x*_1_ − 2.68*x*_2_ − 0.68*x*_3_	0.64 + 3.62*x*_1_ − 2.65*x*_2_ + 2.88*x*_3_
3	l	h	l	− 6.39 + 1.0*x*_1_ − 2.68*x*_2_ − 3.15*x*_3_	− 1.19 + 0.49*x*_1_ + 4.67*x*_2_ + 0.72*x*_3_
4	l	l	h	− 4.35 − 4.56*x*_1_ − 7.97*x*_2_ − 1.81*x*_3_	4.32 − 0.04*x*_1_ + 7.13*x*_2_ − 1.54*x*_3_
5	l	m	h	6.60 − 5.05*x*_1_ − 3.02*x*_2_ + 4.53*x*_3_	2.98 + 3.14*x*_1_ + 3.25*x*_2_ + 4.10*x*_3_
6	l	h	h	− 0.59 − 3.99*x*_1_ − 4.70*x*_2_ + 0.49*x*_3_	6.61 − 2.40*x*_1_ + 3.33*x*_2_ + 5.55*x*_3_
7	m	l	l	5.41 + 0.81*x*_1_ + 3.50*x*_2_ − 9.63*x*_3_	3.60 + 2.34*x*_1_ − 5.49*x*_2_ + 1.98*x*_3_
8	m	m	l	8.68 − 2.02*x*_1_ + 4.81*x*_2_ + 4.94*x*_3_	0.81 + 0.50*x*_1_ − 0.52*x*_2_ + 0.61*x*_3_
9	m	h	l	− 5.59 − 8.59*x*_1_ − 1.69*x*_2_ + 8.38*x*_3_	1.27 + 0.38*x*_1_ + 0.78*x*_2_ − 8.35*x*_3_
10	m	l	h	0.53 + 6.72*x*_1_ − 4.77*x*_2_ + 1.40*x*_3_	3.54 − 2.55*x*_1_ + 6.37*x*_2_ − 4.58*x*_3_
11	m	m	h	1.61 − 0.48*x*_1_ − 4.62*x*_2_ − 3.94*x*_3_	− 7.33 − 4.48*x*_1_ + 7.48*x*_2_ − 1.62*x*_3_
12	m	h	h	− 5.35 + 2.42*x*_1_ + 1.45*x*_2_ − 0.86*x*_3_	2.06 + 1.69*x*_1_ − 1.81*x*_2_ + 0.27*x*_3_
13	h	l	l	1.01 + 1.08*x*_1_ + 0.84*x*_2_ − 1.97*x*_3_	2.44 + 1.38*x*_1_ − 0.03*x*_2_ − 5.30*x*_3_
14	h	m	l	0.65 − 0.05*x*_1_ + 6.56*x*_2_ + 1.32*x*_3_	− 1.62 + 6.66*x*_1_ − 4.24*x*_2_ + 5.29*x*_3_
15	h	h	l	5.49 − 2.56*x*_1_ + 8.23*x*_2_ + 0.61*x*_3_	0.19 − 8.55*x*_1_ − 1.48*x*_2_ − 3.43*x*_3_
16	h	l	h	− 1.12 + 6.32*x*_1_ − 5.46*x*_2_ + 2.12*x*_3_	1.59 − 6.60*x*_1_ + 1.61*x*_2_ + 1.59*x*_3_
17	h	m	h	− 0.77 − 5.47*x*_1_ + 1.52*x*_2_ − 3.11*x*_3_	3.18 + 1.63*x*_1_ − 6.29*x*_2_ + 0.76*x*_3_
18	h	h	h	6.54 − 2.07*x*_1_ + 3.08*x*_2_ + 0.26*x*_3_	8.48 − 0.42*x*_1_ − 7.38*x*_2_ − 0.01*x*_3_

l, low; m, medium; h, high.

**Table 7:** ANFIS rules: antecedents and consequents for labels *healthy tissue* vs. *tumor core* vs. *edema*.

**Rules**	**Antecedent**			**Consequent output**		
	*x*_1_	*x*_2_	*x*_3_	**Healthy tissue**	**Tumor core**	**Edema**
1	l	l	l	1.81 − 7.65*x*_1_ + 0.95*x*_2_ + 6.77*x*_3_	− 9.08 − 3.70*x*_1_ − 1.74*x*_2_ − 0.8*x*_3_	2.78 − 2.27*x*_1_ − 6.57*x*_2_ − 2.14*x*_3_
2	l	m	l	− 9.03 + 9.78*x*_1_ − 1.39*x*_2_ − 5.42*x*_3_	4.68 + 5.49*x*_1_ + 9.52*x*_2_ − 0.96*x*_3_	− 7.5 + 3.32*x*_1_ − 0.45*x*_2_ − 7.67*x*_3_
3	l	h	l	− 5.52 + 8.16*x*_1_ − 2.07*x*_2_ + 9.28*x*_3_	− 7.54 − 1.21*x*_1_ − 1.21*x*_2_ + 1.05*x*_3_	6.42 + 4.75*x*_1_ + 7.47*x*_2_ + 1.18*x*_3_
4	l	l	h	0.27 + 4.71*x*_1_ + 3.29*x*_2_ − 7.62*x*_3_	− 7.47 − 7.21*x*_1_ − 5.22*x*_2_ + 1.1*x*_3_	− 0.75 − 3.98*x*_1_ − 1.03*x*_2_ − 2.55*x*_3_
5	l	m	h	8.84 − 3.03*x*_1_ − 0.36*x*_2_ + 3.16*x*_3_	− 4.36 + 9.16*x*_1_ + 0.86*x*_2_ + 7.07*x*_3_	6.17 − 4.14*x*_1_ + 8.58*x*_2_ + 6.71*x*_3_
6	l	h	h	− 5.52 − 3.86*x*_1_ + 6.22*x*_2_ + 1.08*x*_3_	7.01 + 5.56*x*_1_ − 4.70*x*_2_ + 0.92*x*_3_	− 6.58 + 2.2*x*_1_ + 7.31*x*_2_ − 0.32*x*_3_
7	m	l	l	− 9.95 + 0.98*x*_1_ + 4.61*x*_2_ − 8.78*x*_3_	0.84 + 5.96*x*_1_ + 9.68*x*_2_ − 6.96*x*_3_	− 7.16 − 3.37*x*_1_ + 0.77*x*_2_ − 3.26*x*_3_
8	m	m	l	− 0.31 + 4.71*x*_1_ − 4.95*x*_2_ + 0.76*x*_3_	− 0.77 + 2.49*x*_1_ + 0.75*x*_2_ + 1.16*x*_3_	5.27 + 0.17*x*_1_ − 3.84*x*_2_ + 7.98*x*_3_
9	m	h	l	− 0.27 + 4.92*x*_1_ + 5.51*x*_2_ + 3.29*x*_3_	3.20 + 8.67*x*_1_ + 6.86*x*_2_ − 2.53*x*_3_	− 3.46 + 2.37*x*_1_ + 1.61*x*_2_ + 1.17*x*_3_
10	m	l	h	− 4.55 − 7.51*x*_1_ − 4.14*x*_2_ + 3.74*x*_3_	2.03 − 2.30*x*_1_ + 3.43*x*_2_ − 4.29*x*_3_	− 3.17 + 2.31*x*_1_ − 3.9*x*_2_ + 0.27*x*_3_
11	m	m	h	− 2.05 − 1.97*x*_1_ + 2.14*x*_2_ + 5.06*x*_3_	− 3.83 − 6.64*x*_1_ + 4.45*x*_2_ + 0.43*x*_3_	− 7.17 + 1.79*x*_1_ − 4.93*x*_2_ + 3.9*x*_3_
12	m	h	h	2.39 + 2.98*x*_1_ − 6.54*x*_2_ + 9.83*x*_3_	1.78 + 5.30*x*_1_ − 2.68*x*_2_ − 6.23*x*_3_	− 1.96 + 2.18*x*_1_ − 2.01*x*_2_ − 1.64*x*_3_
13	h	l	l	− 1.26 + 5.83*x*_1_ + 4.58*x*_2_ − 9.45*x*_3_	4.95 + 3.64*x*_1_ − 8.38*x*_2_ − 3.74*x*_3_	− 1.77 + 4.02*x*_1_ + 7.35*x*_2_ + 8.32*x*_3_
14	h	m	l	− 3.86 + 3.64*x*_1_ + 0.19*x*_2_ + 3.45*x*_3_	− 7.61 − 6.79*x*_1_ − 0.89*x*_2_ + 1.39*x*_3_	0.95 − 0.52*x*_1_ − 1.58*x*_2_ + 0.32*x*_3_
15	h	h	l	− 7.14 + 2.25*x*_1_ − 0.26*x*_2_ + 3.68*x*_3_	4.15 − 4.34*x*_1_ + 1.55*x*_2_ − 2.73*x*_3_	− 1.38 + 2.67*x*_1_ + 0.74*x*_2_ + 2.74*x*_3_
16	h	l	h	− 5.40 + 4.37*x*_1_ + 5.72*x*_2_ − 1.24*x*_3_	1.76 − 4.62*x*_1_ + 0.85*x*_2_ − 1.36*x*_3_	2.77 + 2.73*x*_1_ − 3.3*x*_2_ − 0.33*x*_3_
17	h	m	h	1.88 + 2.67*x*_1_ − 0.90*x*_2_ − 2.46*x*_3_	2.77 − 2.6*x*_1_ − 1.57*x*_2_ − 0.98*x*_3_	2.72 + 2.87*x*_1_ − 8.58*x*_2_ + 6.05*x*_3_
18	h	h	h	− 6.65 − 7.27*x*_1_ − 1.03*x*_2_ + 6.36*x*_3_	− 3.96 − 6.99*x*_1_ + 3.7*x*_2_ − 4.89*x*_3_	− 0.89 + 3.67*x*_1_ + 8.98*x*_2_ + 2.29*x*_3_

l, low; m, medium; h, high.

**Figure 8:**
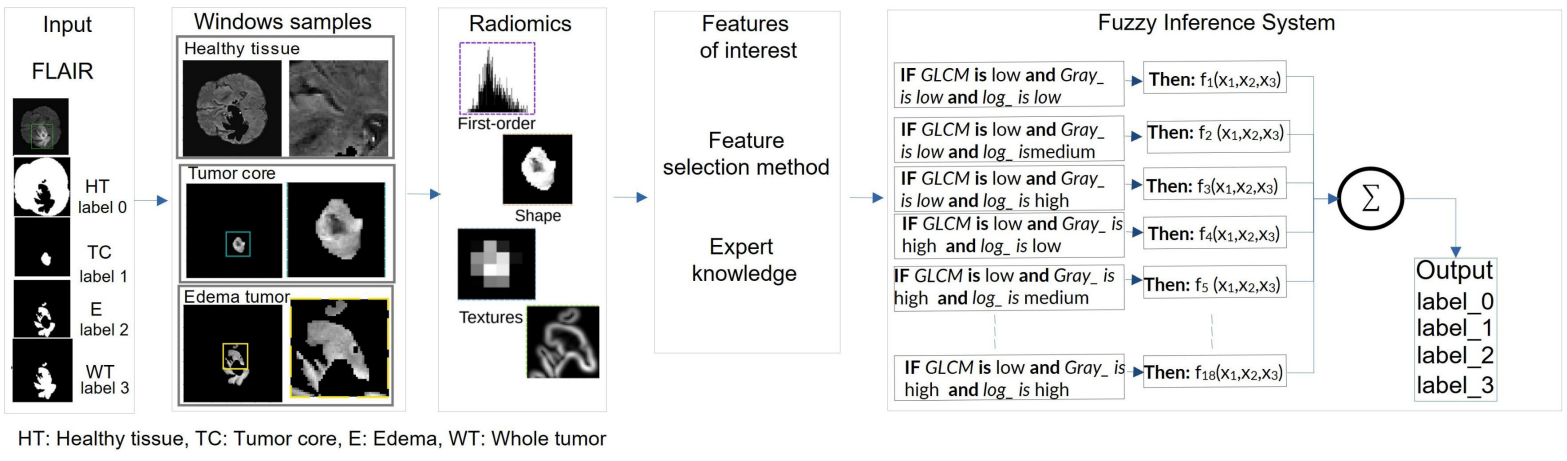
Pipeline of the extraction of the radiomic features for the kernel size and the classification of the radiomic features by ANFIS for the tumor characterization.

The use of decision rules, as shown in Figure 8, illustrates the interpretability of the classification process for the experiments using the ANFIS model with only 18 fuzzy rules to classify brain tissue. The current study shows the use of automatic feature selection, but the proposal is open to feature selection with expert knowledge. Tables 6, 7 show the learned parameters, where each input variable is assigned linguistic labels based on expert knowledge. Fuzzy rules evaluate the classification by processing the input features through a weighted combination of rules to determine the final output.

The output of the ANFIS model generates the antecedent and consequent parameters. These parameters correspond to the inference process of decision rules that weights the classified label (e.g., healthy tissue, tumor core, edema). The model applies the IF-THEN rules for each input case, where each rule is associated with a degree of activation based on how well the input features match the linguistic conditions. For example, the first fuzzy rule in Experiment 1 evaluates whether the input corresponds to healthy tissue (output 0) or a whole tumor (output 1). Each rule assigns a weight or degree of confidence to potential outputs depending on how well the input characteristics (e.g., feature values) satisfy the conditions of the rule.

The system then aggregates these weighted outputs across all 18 rules using a weighted average. This process is known as rule aggregation, where the contributions of each rule are combined to generate the final decision. The output with the highest cumulative weight is selected as the final classification label. In this case, the model decides whether to classify the tissue as healthy (label_0) or tumor (label_3) based on the overall strength of the activated fuzzy rules.

## 5 Discussion

This study introduces a novel framework for tumor characterization in MRI, utilizing the combination of ANFIS and a feature selection approach. Integrating these methods aims to enhance the precision and efficiency of tumor analysis, addressing current challenges in medical imaging. The proposed model demonstrated good segmentation performance using the BraTS2020 dataset, achieving DICE Scores of 99.90% for healthy tissue, 82.94% for tumor core (TC) and 76.06% for edema. These results are consistent with those reported by Baid et al. (2020), who achieved DICE Scores of 92% for the whole tumor, 90% for tumor core and 81% for the enhancement tumor. Similarly, Akbar et al. (2021) reported DICE Scores of 78.02%, 80.73% and 89.07% for the enhancing tumor, tumor core, and whole tumor, respectively, using a 3D U-net architecture. Although the DICE Score for edema (76.06%) is lower than other lesions, primarily due to its diffuse nature, structural complexity, and inherent difficulty in distinguishing edema from tumor infiltration, our model demonstrates the ability to identify these challenging structures.

Several studies utilizing ANFIS classification mechanisms for BT are showing promising results in the identification of brain tissue. For example,the proposal by Shankar et al. (2020) presents an ANFIS method to classify brain MRI into benign and malignant tumors with an accuracy of 96.23%. Mathiyalagan and Devaraj (2021) and Nagarathinam and Ponnuchamy (2019), propose an ANFIS-based method to classify tumors. It achieves a rate of recognition of gliomas greater than 98% accuracy. However, while these developments demonstrate high performance in their outcomes, they do not elucidate how the results can be interpreted. Such proposals utilizing these ANFIS models fail to explain in their methodology how to derive output decisions based on their input data. This approach, based on ANFIS and radiomics, integrates fuzzy decision rules that offer valuable insights into the diagnostic process. The ANFIS-based model achieved an accuracy of 95.20% in the healthy tissue versus whole tumor experiment, and 92.14% in the classification of healthy tissue, tumor core and edema, closely aligned with the performance of other machine learning classifiers. However, this methodology addresses a critical gap: It emphasizes a comprehensive mechanism to explain how radiomic features contribute to the decision-making process, improving interpretability.

The challenge of balancing performance and interpretability is particularly evident in ANFIS models. Increasing the number of radiomics features has been shown to improve performance, but this often leads to an exponential increase in rule complexity, making models less interpretable and difficult to validate clinically. Previous studies have attempted to reduce the number of rules, but have often resulted in impractical rule sets that do not meet clinical needs. For example, Hien et al. (2022) reduced the number of rules in the ANFIS models without achieving low rules to facilitate interpretability. This feature selection approach is consistent with studies that emphasize the role of radiomic features in improving classification accuracy while balancing model complexity (Tahosin et al., 2023; Lefkovits et al., 2017; Khanna et al., 2024). This study overcomes these challenges by minimizing the complexity of the rule without sacrificing the accuracy of the classification. A streamlined feature selection process ensures that only the most informative features are used, resulting in a concise set of interpretable rules capturing the essence of diagnostic patterns. This approach reduces computational overhead and increases transparency, allowing clinicians to understand and validate the model's reasoning. Furthermore, by linking radiomics features to diagnostic assistance outcomes, the generated fuzzy rules provide a framework for interpreting the classification process.

## 6 Conclusion

This study presents an integrated system that combines a CNN-based segmentation model with an interpretability framework for a clinically relevant and accurate characterization of tumor diagnosis assistance using MRI data. The model achieved high precision in the segmentation stage, with average DICE Scores of 99.90% for healthy tissue, 82.94% for tumor core (TC), and 76.06% for edema. These results demonstrate the effectiveness of the model in accurately identifying relevant tissue regions, particularly healthy tissue and the tumor core. However, edema segmentation exhibited more variability due to its diffuse nature, structural complexity, and challenges posed by differential diagnoses.

Following segmentation, an interpretability framework was introduced, integrating radiomics-based feature extraction with an ANFIS model to interpret classification results through fuzzy rules. Initially, 218 radiomic features were extracted and a feature selection process was applied using Decision Tree and Random Forest to identify the most relevant features. These selected features were evaluated in two experiments: the first distinguishing between healthy tissue and whole tumor, and the second differentiating among healthy tissue, tumor core, and edema. The results demonstrated high accuracy, achieving over 95% in the two-class experiment and 92% in the three-class experiment. The combination of radiomics and ANFIS proved effective in delivering interpretability through 18 decision rules generated from the three most relevant radiomic features.

However, some limitations were observed. As the number of input features increases, the number of rules in ANFIS grows, which can reduce the system interpretability. The proposed framework was also tested exclusively on FLAIR images, highlighting the need to extend the analysis to other MRI modalities. Future research should address these limitations by exploring techniques to manage the growing number of fuzzy rules, such as rule reduction strategies or hybrid methods that balance interpretability and accuracy. Efforts should also include integrating additional MRI modalities and extending to 3D imaging to provide a more comprehensive and clinically relevant framework. In doing so, radiomics for feature extraction and ANFIS for classification can be further validated as promising approaches to improve both the accuracy and interpretability of BT diagnosis and characterization.

## Data Availability

Publicly available datasets were analyzed in this study. This data can be found at: https://www.med.upenn.edu/cbica/brats2020/data.html.
